# Single-cell RNA sequencing of cultured human endometrial CD140b^+^CD146^+^ perivascular cells highlights the importance of in vivo microenvironment

**DOI:** 10.1186/s13287-021-02354-1

**Published:** 2021-05-29

**Authors:** Dandan Cao, Rachel W. S. Chan, Ernest H. Y. Ng, Kristina Gemzell-Danielsson, William S. B. Yeung

**Affiliations:** 1 Shenzhen Key Laboratory of Fertility Regulation, Reproductive Medicine Center, The University of Hong Kong - Shenzhen Hospital, Shenzhen, China; 2 Department of Obstetrics and Gynaecology, LKS Faculty of Medicine, The University of Hong Kong, Pokfulam, Hong Kong, SAR China; 3 Department of Women’s and Children’s Health, Division of Obstetrics and Gynecology, Karolinska Institutet and Karolinska University Hospital, Solna, Sweden; 4 Department of Obstetrics and Gynecology, The University of Hong Kong, Pokfulam, Hong Kong, SAR China

**Keywords:** Endometrial perivascular cells, Single-cell RNA sequencing, Heterogeneity, Microenvironment

## Abstract

**Background:**

Endometrial mesenchymal-like stromal/stem cells (eMSCs) have been proposed as adult stem cells contributing to endometrial regeneration. One set of perivascular markers (CD140b&CD146) has been widely used to enrich eMSCs. Although eMSCs are easily accessible for regenerative medicine and have long been studied, their cellular heterogeneity, relationship to primary counterpart, remains largely unclear.

**Methods:**

In this study, we applied 10X genomics single-cell RNA sequencing (scRNA-seq) to cultured human CD140b^+^CD146^+^ endometrial perivascular cells (ePCs) from menstrual and secretory endometrium. We also analyzed publicly available scRNA-seq data of primary endometrium and performed transcriptome comparison between cultured ePCs and primary ePCs at single-cell level.

**Results:**

Transcriptomic expression-based clustering revealed limited heterogeneity within cultured menstrual and secretory ePCs. A main subpopulation and a small stress-induced subpopulation were identified in secretory and menstrual ePCs. Cell identity analysis demonstrated the similar cellular composition in secretory and menstrual ePCs. Marker gene expression analysis showed that the main subpopulations identified from cultured secretory and menstrual ePCs simultaneously expressed genes marking mesenchymal stem cell (MSC), perivascular cell, smooth muscle cell, and stromal fibroblast. GO enrichment analysis revealed that genes upregulated in the main subpopulation enriched in actin filament organization, cellular division, etc., while genes upregulated in the small subpopulation enriched in extracellular matrix disassembly, stress response, etc. By comparing subpopulations of cultured ePCs to the publicly available primary endometrial cells, it was found that the main subpopulation identified from cultured ePCs was culture-unique which was unlike primary ePCs or primary endometrial stromal fibroblast cells.

**Conclusion:**

In summary, these data for the first time provides a single-cell atlas of the cultured human CD140b^+^CD146^+^ ePCs. The identification of culture-unique relatively homogenous cell population of CD140b^+^CD146^+^ ePCs underscores the importance of in vivo microenvironment in maintaining cellular identity.

**Supplementary Information:**

The online version contains supplementary material available at 10.1186/s13287-021-02354-1.

## Background

The uterine cavity is lined by the endometrium, which is shed off and regenerates in each menstrual cycle. This remarkable physiological remodeling occurs about 400 times in a woman’s reproductive life. Adult stem cells are undifferentiated cells found throughout the body after development. They proliferate and differentiate to replenish dying cells and to regenerate damaged tissues. In endometrium, stromal stem cells was firstly identified as clonogenic cells with multiple lineage differentiation potential in vitro [1]. Endometrial stromal stem cells exhibited properties similar to that of mesenchymal stem cells (MSCs) in other tissues in terms of clonogenicity, fibroblast-like morphology, surface markers’ phenotype, and multipotency. Thus, they are called endometrial mesenchymal-like stem cells (eMSCs).

MSCs, including eMSCs, exhibit great differentiation potential and immunomodulation ability enabling them for cell therapeutic use. Among MSCs, eMSCs are the only one that can easily be obtained from women each month without use of analgesics. A woman can use her own eMSCs for therapy when needed. Therefore, eMSCs have been tested as an alternative source for cell therapies. Transplantation of human eMSCs to mouse and primate models of Parkinson’s disease significantly increases the dopamine level when compared to sham transplanted controls [2, 3]. In addition, eMSCs have been also studied for regenerative medicine in other diseases including diabetes, cardiac diseases, and cartilage injury [4].

Lessons from clinical trials of MSCs show that differences in preparation of MSCs such as culture and expansion method affect clinical trial outcome of MSCs [5]. For instance, bone marrow-derived MSCs exhibit cellular heterogeneity during expansion in vitro [6], and MSCs from different clones exhibited substantial variation in differentiation potential [7]. Cellular heterogeneity of Wharton’s jelly MSCs and limited heterogeneity of MSCs from umbilical cord have been reported by other studies [8, 9]. However, little is known about the heterogeneity of eMSCs.

In addition to utilizing eMSCs as a great source for cell transplantation, researchers also proposed the in situ activation of eMSCs to treat endometrial disorders [10–12]. However, the regenerative ability of eMSCs was merely confirmed according to experiments based on cultured endometrial perivascular cells (ePCs). Their existence in vivo remains to be explored. By isolating ePCs using two eMSC-enriching markers CD140b and CD146 [13], Spitzer et al. obtained transcriptomic expression of primary ePCs and cultured ePCs [12]. However, this dataset was conducted in bulk level which did not account for the heterogeneity of the cells.

The recent development of single-cell RNA sequencing (scRNA-seq), which combines single-cell isolation techniques with RNA-seq, creates an opportunity to study the transcriptomes of individual cells enabling clear distinctions between subpopulations, and thorough assessment of gene transcripts in an unbiased manner [14]. Regarding questions raised above, in this study, we aim to characterize the gene expression of the cultured CD140b^+^CD146^+^ ePCs from menstrual and secretory stages at single-cell resolution. We revealed similar main subpopulation and small DNA-damag-induced senescent subpopulation in both cultured secretory and menstrual ePCs. Linking analysis to primary PDGFRB^+^MCAM^+^ (CD140b^+^CD146^+^) endometrial stromal cells classified the main subpopulation as culture-unique and small DNA-damage-induced senescence subpopulation as stromal fibroblasts. Our study for the first time fills the knowledge gap on understanding the heterogeneity and cell identity of cultured ePCs at single-cell level which highlights the importance of in vivo microenvironment in maintaining the cellular profiles.

## Methods

### Human endometrial samples

Menstrual phase samples (*n*=3) were collected by endometrial aspiration from three women with regular menstrual cycles (median age 32; range 31–40 years) attending the infertility clinic on days 2–3 of their menstrual cycle (Table S1). Full thickness endometrial samples were collected from women with regular menstrual cycles (median age 50; range 49–52 years) who underwent total abdominal hysterectomy for benign non-endometrial pathologies (Table S1). They had not taken hormonal therapy in the past 3 months before the surgery. Based on histology of endometrial sections, experienced pathologists diagnosed that the full thickness endometrial samples (*n* = 3) collected were at the secretory phase of the menstrual cycle.

### Isolation of endometrial cells

Endometrial tissues were minced into 1 mm^3^ pieces and dissociated in phosphate-buffered saline (PBS) containing collagenase type III (0.3 mg/ml, Worthington Biochemical Corporation, Freehold, NJ, USA) and deoxyribonuclease type I (40 μg/ml, Worthington Biochemical Corporation) in a shaking water bath for 60 min at 37^o^C [15]. At 15-min interval, the digests were pipetted vigorously and dissociation was monitored microscopically. Trypan blue was used to assess cell viability. After two rounds of digestion, the dispersed cells were filtered through 40-μm sieves (BD Bioscience, San Jose, CA, USA), loaded onto Ficoll-Paque (GE Healthcare, Uppsala, Sweden) for removal of red blood cells, cell debris, and cell clumps by centrifugation. Anti-CD45 antibody-coated Dynabeads (Invitrogen, Waltham, MA, USA) were used to eliminate leukocytes. Stromal cells were negatively selected using microbeads coated with antibody against epithelial cell marker CD368 (EpCAM) (Miltenyi Biotech, Bergisch Gladbach, Germany). Freshly purified stromal cells were plated at 2500 cells/cm^2^ onto 100-mm dishes coated with fibronectin (1 mg/ml, Invitrogen). They were cultured in growth medium containing 10% FBS (ThermoFisher Scientific, Waltham, MA, USA), 1% penicillin (ThermoFisher Scientific), and 1% L-glutamine (ThermoFisher Scientific) in DMEM/F12 (Sigma-Aldrich, St Louis, MA, USA). The stromal cells were expanded in culture for 7–14 days in a humidified carbon dioxide incubator at 37 °C to reach 80% confluence for downstream eMSC enrichment. The culture medium was changed every 7 days.

### Magnetic bead selection of endometrial mesenchymal stem-like cells

Isolation of eMSCs (CD140b^+^CD146^+^ cells) was conducted with two separate positive magnetic bead selections [16]. Stromal cells were incubated with phycoerythrin (PE)-conjugated anti-CD140b antibody at 4 °C for 45 min. The cells were then incubated with anti-mouse IgG1 magnetic microbeads (Miltenyi Biotech) at 4^o^C for 15 min. The CD140b^+^ cells were collected using the Miltenyi columns with a magnetic field and cultured for 7 to 10 days in growth medium to allow degradation of the microbeads during cell expansion. The CD140b^+^ cells were then trypsinized and incubated with anti-CD146 microbeads (Miltenyi Biotech) at 4^o^C for 15 min. The CD140b^+^CD146^+^ cells were collected and used for single-cell RNA sequencing.

### Dual Immunofluorescence

Double immunofluorescent staining was performed to evaluate the phenotypic of CD140b^+^CD146^+^ cells. Some of the cells after magnetic microbeading against CD140b and CD146 were plated at low seeding density of 10–30 cells/cm^2^ on fibronectin-coated 12-well plates and culture for 3–4 days. Cells were fixed with 4% paraformaldehyde for 20 min. Permeabilization was performed using 0.1% Triton-X 100 for 10 min and blocked with 2% BSA for 30 min. Cells were incubated with primary antibodies; anti-human CD140b (1:200; R&D Systems) and anti-human CD146 (1:100; Abcam, Cambridge, UK) antibodies at 4 °C overnight. The following day, cells were incubated with the secondary antibodies: donkey anti-mouse antibodies conjugated with Alexa Fluor 564 (1:200; ThermoFisher Scientific) and goat anti-rabbit antibodies conjugated with Alexa Fluor 488 (1:200; ThermoFisher Scientific). The cell nuclei were detected by DAPI (ThermoFisher Scientific). All washing steps were performed with PBS and conducted at room temperature unless specified. Images were captured using a Carl Zeiss LSM inverted confocal microscope and a Zeiss LSM Zen 2010 software (Carl Zeiss, Munich, Germany) at the Centre for PanorOmics Sciences (CPOS) Imaging and Flow Cytometry Core, The University Of Hong Kong.

### Single-cell library construction and RNA sequencing

Single-cell library construction and RNA sequencing (scRNA-seq) were performed at the Genomics Core, Centre for PanorOmic Sciences (CPOS), The University of Hong Kong. Single-cell encapsulation and cDNA libraries were prepared by the Chromium™ Single Cell 3’ Reagent Kits v2/v3 and the Chromium™ Single Cell A/B Chip Kit. Libraries were sequenced on an Illumina NovaSeq 6000 instrument using paired-end 151 bp.

### Mapping of sequencing reads to human transcriptomes and original cells

High-quality sequencing reads from each sample were separately mapped to the human reference genome and transcriptome (GRCh38-3.0.0) using the STAR aligner [17] in the 10X Genomics cellranger pipeline (v3.0.2). Aligned reads were filtered for valid cell barcodes and unique molecular identifier (UMI) during cellranger count process. The cells were called by a new algorithm EmptyDrops [18] which is introduced in cellranger v3 pipeline.

### Preprocessing of scRNA-seq data

The generated files from cellranger v3 pipeline were loaded into R package Seurat (version 3.2.3) [19, 20] to produce the gene-cell matrix for each sample. Object for each sample was created by filtering cells with detected genes < 200 and genes with expression < 3 cells. Gene filtering was further performed to retain those genes that commonly detected by both versions of chemistry reagents. Sample-level quality control was performed based on several conditions as shown in the supplementary file. Cells from the same menstrual stage were merged together for downstream analysis.

### Defining highly variable genes

To define highly variable genes (HVGs), we firstly normalized the data using the Seurat function NormalizeData with method “logNormalize”. We then applied the method “vst” of Seurat function FindVariableGenes to identify the top 3000 HVGs for subsequent analysis.

### Batch-effect correction

Batch integration was initially evaluated for menstrual and secretory samples separately using 3 methods including Fast mutual nearest neighbors (fastMNN) (in package batchelor (version 1.2.4)) [21], Harmony (version 1.0) [22], and Seurat v3 [19, 20] recommended by two benchmark studies [23, 24]. Briefly, data normalization and HVGs defining were performed firstly for each batch (donor). FastMNN was applied using RunFastMNN function of SeuratWrapper package by setting the order as true. Harmony was applied using RunHarmony function of SeuratWrapper package after PCA dimensional reduction. For Seurat v3, anchors across batches were identified using the FindIntegrationAnchors and the data were finally integrated using the IntegrateData function of the Seurat package. The assay “integrated” was used for downstream analysis including data scaling, dimensional reduction and clustering. For secretory and clonogenic cells, batch effect was removed using Harmony given its superiority in integrating samples either with shared subpopulation(s) or with distinct cell types.

### kBET acceptance score to quantify batch-effect correction

kBET acceptance scores were calculated using the pipeline from Buttner et al. [25] to assess the batch-effect correction performance. The metric was calculated using the low-dimensional embeddings matrices of each batch-correction method. The kBET acceptance score was calculated for secretory ePCs and menstrual ePCs.

### Silhouette score to quantify batch-effect correction and clustering performance

The silhouette score of each cell was calculated based on secretory or menstrual ePCs, or different clusters of ePCs with the function “silhouette” from the R package cluster (version 2.1.0). Both the mean and distribution of silhouette scores were used for selection of the batch-correction methods and clustering resolution.

### Data scaling, cell cycle phase classification, and cell cycle effect removal

Data scaling was performed using the ScaleData function of the Seurat package. When scaling the data, five factors were considered: total UMI count per cell, number of genes per cell, percent of mitochondrial reads per cell, cell cycle effect, and donor age. To classify the cell cycle phase of each cell, we firstly assigned a score to each cell based on its expression of G2/M and S phase markers [26] using the CellCycleScoring function in the Seurat package [19, 20]. Cells were assigned to the G2/M or S phase based on their expression score, while cells expressing neither were likely not cycling and were assigned to the G1 phase. To remove the cell cycle effect, the S scores and the G2/M scores were used to regress out cell cycle effect using ScaleData.

### Dimensionality reduction

Dimensionality reduction was performed after data scaling. Principal component analysis (PCA) was performed to reduce the data to the top 50 PCA components.

### Clustering

We conducted a graph-based clustering approach. Firstly, a *K*-nearest neighbor (KNN) graph was constructed based on the Euclidean distance in the integrated assay, with refined edge weights between any two cells based on Jaccard similarity using the FindNeighbors function of the Seurat package. Next, the Louvain algorithm was applied to cluster the cells using the FindClusters function with resolution of 0.1 of the Seurat package. We visualized the clusters on a 2D map produced with the Uniform Manifold Approximation and Projection (UMAP).

### Post-clustering quality control

Clusters with low quality (low UMI, low number of cells) were filtered out. Data analyses were re-performed on the remaining cells until no low-quality clusters were present in the dataset. The finalized clusters were retained and used for visualization and gene expression inspection.

### Correlation analysis

We quantified the correlation of single-cell clusters based on average gene expression using the *cor* (method pearson) function in R (4.0.3).

### MetaNeighbor analysis

MetaNeighbor analysis was performed using the the R function MetaNeighbor (version 1.10.0) with default settings [27]. The AUROC (area under the receiver operating characteristic) scores produced by the MetaNeighbor analysis indicate the degree of correlation between cell groups. An AUROC score of 0.5 means that the probability of correct assignment of a cell’ identity in a binary classification is the same as random guessing.

### Differential expression of gene signatures

For each cluster/subpopulation, we used the Wilcoxon rank-sum test to find gene that had significantly different expression when compared to the remaining clusters using the FindAllMarkers function in the Seurat package. Only positive markers were considered. Genes with log fold change larger than 0.25 and Bonferroni correction *p* values less than 0.05 were retained and used for further analysis.

### GO enrichment analysis

Upregulated genes (UGs) for each finalized sub-cluster were firstly identified in RNA assay using the FindAllMarkers function of the Seurat package. Log fold change was set as 0.25. The generated UGs were subjected to GO enrichment analysis using R package clusterProfiler (version 3.18.0) [28].

## Results

### Overview of the single-cell RNA sequencing

**Fig. 1:**
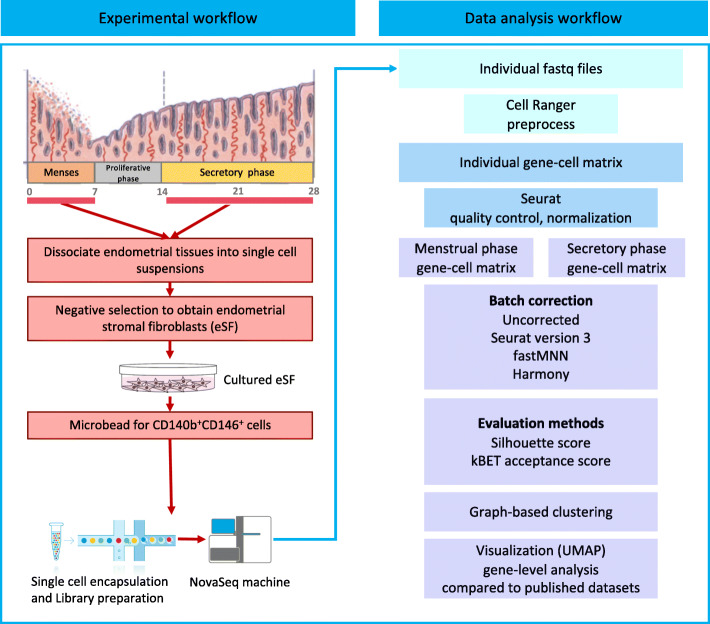
Overview of the experimental procedure. Schematic representation of the experimental and bioinformatics analysis workflow. EPCs were enriched by CD140b and CD146 from cultured primary stromal cells isolated from endometrium of 6 female donors (3 secretory phase and 3 menstrual phase) with regular menstrual cycle and normal endometrium. Enriched ePCs were directly subjected to scRNA-seq. Generated sequencing data were analyzed as outlined in the bioinformatics workflow

As summarized in Fig. 1, endometrial aspirates from the menstrual phase of three women and full-thickness endometrium from the secretory phase of three women were used in this study (Table S1). After enzyme dispersion of the tissues, the CD140b^+^CD146^+^ endometrial perivascular cells (ePCs) were obtained from in vitro expanded endometrial stromal cells by serial magnetic microbeading [11, 16]. Phenotypic study of the ePCs showed their positive expression on both CD140b and CD146 (Figure S1). These cells were subjected to scRNA-seq on a 10X genomics platform. ScRNA-seq libraries for each sample were constructed independently (Table S1). Comprehensive bioinformatics analysis pipeline was then applied to analyze the sequencing data both technically and biologically (Fig. 1).

We obtained around 5 × 10^8^ sequence reads for each sample, with comparable exonic mapping rate (Figure S2A), but variable cell number (Figure S2B) across them. The variation observed in cell number was due to the use of different reagent versions for single-cell encapsulation and library preparation for samples (Table S1). Quality control was performed on metrics including distribution of number of UMIs, number of detected genes, percentage of mitochondrial UMIs, expression of MSC definition markers, perivascular cell markers, smooth muscle cell markers, and stromal fibroblast cell markers (Figure S2C, S2D, S2E). The results showed that one of the menstrual samples M3 exhibited low quality with low UMI and high mitochondria reads indicating its apoptotic identity (Figure S2C). Furthermore, M3 expressed low level of MSC markers and high level of stromal fibroblast markers suggesting its loss of MSC identity (Figure S2D, S2E). The average gene expression correlation analysis between samples also identified M3 as an outlier (Figure S2F). We further assigned cell cycle phases to each cell according to cell cycle gene expression. Distribution analysis of cell cycle phases showed that G1 cells were significantly enriched in M3 (chi-squared test, *p* < 0.0001) supporting its senescent/apoptotic identity (Figure S2G). Based on these results, M3 was excluded from the downstream analysis (Fig. 1). Cells in the retained samples were filtered out if their number of detected genes were lower than 200. The final resulting cell number for downstream analysis is 19,345 (15,889 for cultured secretory ePCs, 3456 cultured menstrual ePCs).

### Batch effects and batch-effect corrections

**Fig. 2.**
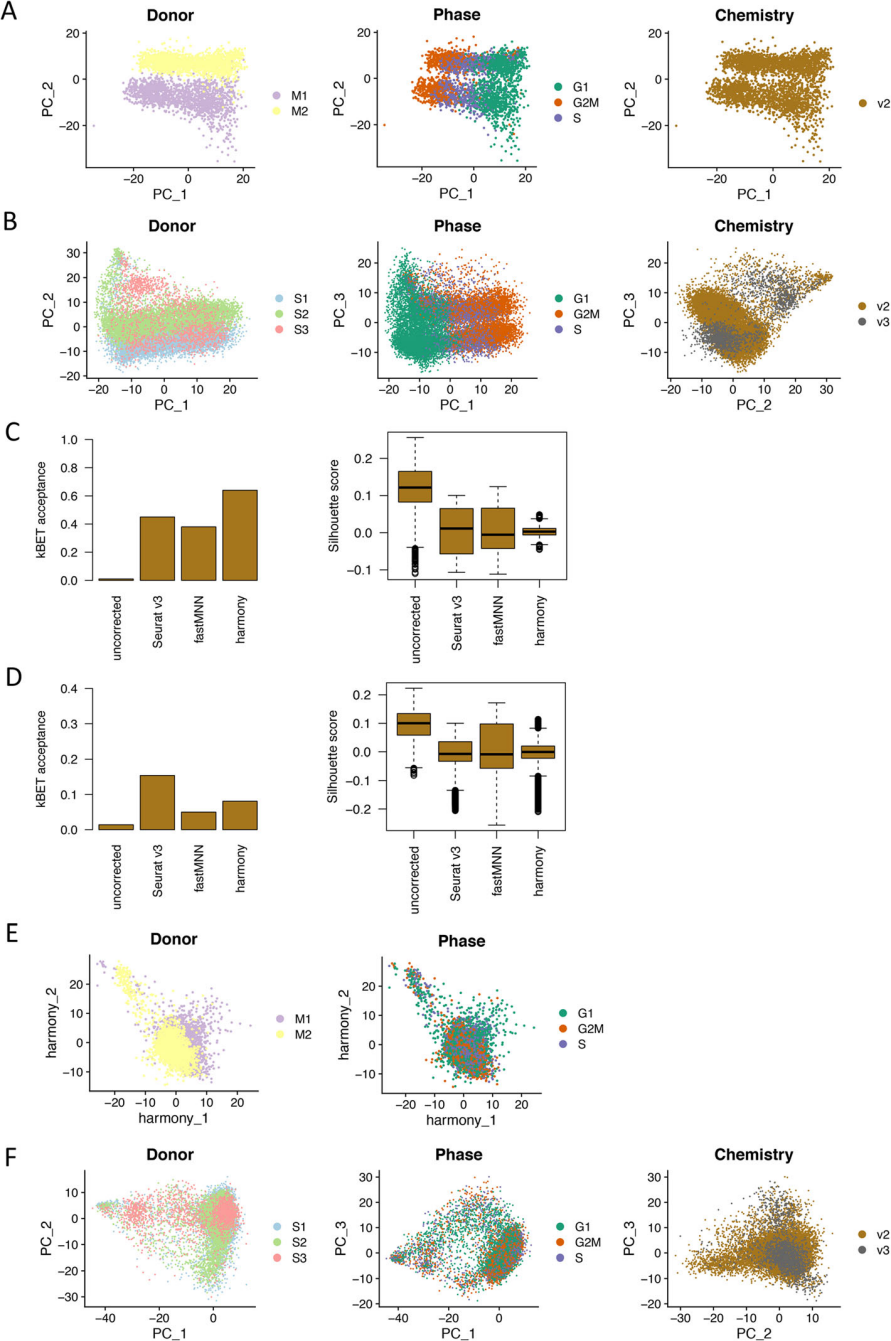
Batch effects and batch-effect correction. a Dimensional reduction plot on the first two principal components showing the distribution of menstrual ePCs color coded by donor, cell cycle phase, and chemistry reagent version. Clear donor effect and cell cycle effect were observed. b Dimensional reduction plot on the first three principal components showing the distribution of secretory ePCs color coded by donor, cell cycle phase, and chemistry reagent version. Clear donor effect, cell cycle effect, and chemistry reagent version effect were observed. c Evaluation on batch-effect correction methods using kBET acceptance score (left panel) and Silhouette score (right panel) on menstrual ePCs. All three methods Seurat v3, fastMNN, and harmony removed the batch effects to different extents when compared to the uncorrected data. Harmony ranked the best by achieving the highest kBET acceptance score and narrowest Silhouette score. d Evaluation on batch-effect correction methods using kBET acceptance score (left panel) and Silhouette score (right panel) on secretory ePCs. All three methods Seurat v3, fastMNN and harmony removed the batch effects to different extents when compared to the uncorrected data. Seurat v3 ranked the best by achieving the highest kBET acceptance score and lowest Silhouette score. e Dimensional reduction plot on the first two harmony dimensions showing the distribution of cells color coded by donor after batch effect correction and cell cycle phase after cell cycle regression. f Dimensional reduction plot on the first two principal components showing the distribution of cells labeled by donor after batch effect correction and cell cycle phase after cell cycle regression

To identify the major variance in the cultured ePC population at single-cell level, principal component analysis (PCA) based on normalized gene expression matrix was conducted separately for secretory samples and menstrual samples. The results showed large donor effect, cell cycle effect for menstrual and secretory samples (Fig. 2a, b) which were consistently observed in other single-cell studies on MSCs [8, 9, 29]. Additionally, chemistry effect was also observed for secretory samples among which different chemistry reagents were used (Fig. 2b). To study biological changes of interest in single-cell data, these technical effects should be removed. Several studies have benchmarked different algorithms of batch-effect correction for different sample scenarios [23, 24]. We chose the top three Seurat v3 [19, 20], fastMNN [21], and Harmony [22] for evaluation in our study. We hypothesized that cultured ePCs in our study from the same menstrual cycle phase should have similar cell composition. Quantitative metrics including kBET acceptance score [25] and Silhouette score were applied to select the method with best performance. The results showed that Harmony was the best method in batch correction for menstrual samples (Fig. 2c), while Seurat v3 was the best one for secretory samples (Fig. 2d) which produced highest kBET acceptance score and lowest Silhouette scores. In our study, secretory samples were processed using two versions of chemistry reagents which manifested different cell number and gene coverage. Seurat v3 ranked the top for secretory samples which is consistent with previous report on the advantage of Seurat v3 in integrating datasets of biologically similar samples from different sequencing platforms [24]. After Harmony and Seurat v3 correction, donor and chemistry effects were successfully removed in menstrual and secretory samples (Fig. 2e, f). Cell cycle effect was also successfully removed by linear regression method implemented in the Seurat package (Fig. 2e, f) [19, 20].

### Limited cellular heterogeneity revealed by single-cell analysis for cultured menstrual ePCs

**Fig. 3:**
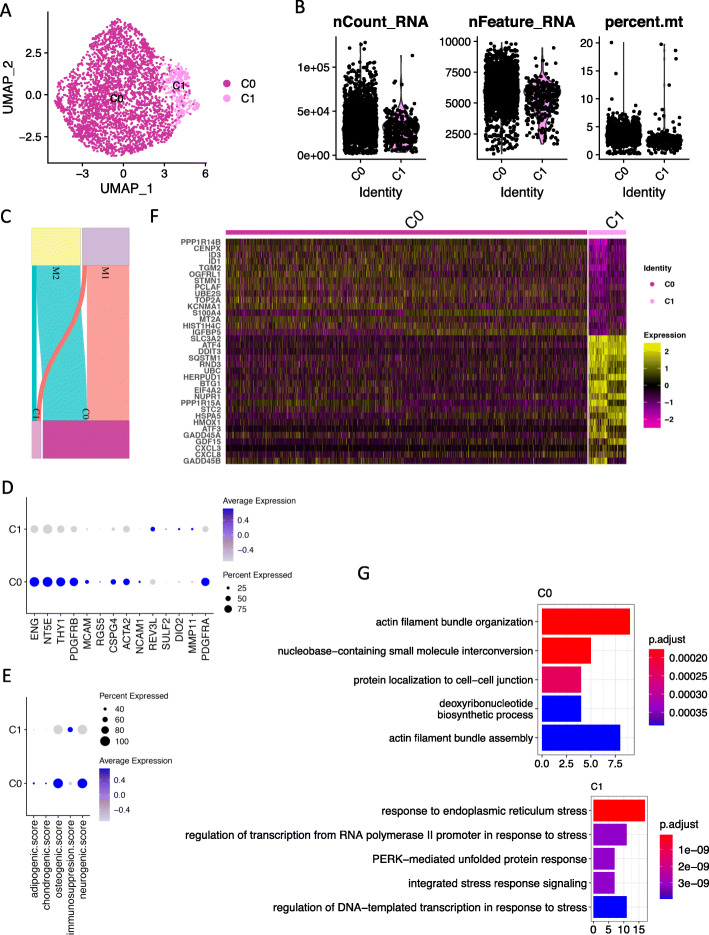
scRNA-seq reveals two sub-clusters in cultured menstrual ePCs. **a** Uniform Manifold Approximation and Projection (UMAP) plot of the cellular sub-clusters. Single cells are color coded by cluster annotation. **b** Violin plot showing the distribution of number of UMIs, number of genes, and percentage of mitochondria reads of single cells in each sub-cluster. **c** Sankey diagram showing the contribution of cells from each sample to the sub-clusters. **d** Dot plot showing the expression of MSC markers, perivascular cell markers, smooth muscle cell markers, and stromal fibroblast cell markers across sub-clusters. Circle size indicates the percentage of cells in which the gene expression was detected. Fill color depicts the averaged normalized expression level of all cells within that sub-cluster. **e** Dot plot showing the score value of adipogenic, chondrogenic, osteogenic, and neurogeneic differentiation and immunomodulation potential across sub-clusters. The score for each cell for each term was firstly calculated by averaging the normalized-expression value of markers in each term. **f** Heat map of top differentially expressed genes between sub-cluster C0 and sub-cluster C1. Sub-clusters were clearly separated. **g** Top significant enriched biological processes from gene ontology analysis based on upregulated genes identified in each sub-cluster. P.adjust, adjusted *p* value, value on *x* axis is the enrichment fold

After cell cycle regression and batch-effect correction, candidate population clustering by a shared nearest neighbor (SNN) graph-based approach revealed 3 subpopulations for the 3456 cultured menstrual ePCs (Figure S3A). Post-clustering quality control showed that one cluster of cells demonstrated extremely low number of UMIs, low number of genes but high mitochondrial reads implying their apoptotic identity (Figure S3B). These low-quality cells were excluded from analysis. Clustering process was re-performed on the remaining cells (3,223) and two stable high-quality clusters (C0, C1) were identified (Fig. 3a, b) under the guidance of Silhouette score (Figure S3C). The contribution of each sample to each cluster is shown in Fig. 3c.

To understand the biological characteristics of the identified C0 and C1, we firstly checked the expression of known MSC markers (ENG, NT5E, THY1), perivascular cell markers (PDGFRB, MCAM, RGS5, CSPG4), smooth muscle cell markers (ACTA2, ALPL, NCAM1), and stromal fibroblast cell markers (REV3L, SULF2, DIO2, MMP11, PDGFRA). The results showed that C0 expressed higher of MSC markers, perivascular cell markers, and smooth muscle cell markers, while C1 expressed higher stromal fibroblast cell markers of REV3L, DIO2, and MMP11 (Fig. 3d, Figure S3D). Interestingly, the canonical fibroblast marker PDGFRA was preferentially expressed in C0 (Fig. 3d, Figure S3D). These results indicated C0 might be a mixture cell population. However, independent clustering on C0 could not further identify clear cell clusters representing MSCs, perivascular cells, smooth muscle cells, and stromal fibroblast cells (data not shown). MSC are a class of cells with multipotency and immunomodulation ability. We further checked the lineage differentiation ability defined by lineage marker expression (Table S2). C0 exhibited higher osteogenic and neurogenic differentiation ability, while C1 exhibited higher immunomodulation ability (Fig. 3e, Figure S3E).

In addition to the investigation on known cell markers, we also identified upregulated genes (UGs) in sub-clusters C0 and C1 using FindAllMarkers function in Seurat package with defined cutoff. The top UGs in C0 and C1 including proliferative genes UBE2S, TOP2A, HIST1H4C, and stress-related genes ATF4, DDIT3, GDF15, clearly separated the two clusters (Fig. 3f). Gene ontology (GO) enrichment analysis on UGs in C0 identified biological processes including actin filament bundle organization and assembly, while UGs in C1 enriched out biological processes relating to responses to stress (Fig. 3g). The GO enrichment results together with high expression of stress-related genes and senescence-associated inflammatory mediators in C1 suggested the senescent cell identity of this cell subpopulation (Fig. 3f, g). Indeed, the genes involved in DNA-damage-induced senescence preferentially expressed in C1 cells (Figure S3F).

In summary, for the cultured menstrual ePCs, we revealed a main subpopulation C0 (2917 cells) which manifested characteristics of MSC, perivascular cells, smooth muscle cells, and stromal fibroblast cells by expressing respective known cell identity markers. We also identified a small subpopulation C1 (306 cells) as a group of senescent cells. Whether C1 was a technical induced subpopulation by mechanistic single-cell isolation or a natural subpopulation emerged from culturing required further investigation.

### Limited cellular heterogeneity revealed by single-cell analysis for cultured secretory ePCs

**Fig. 4:**
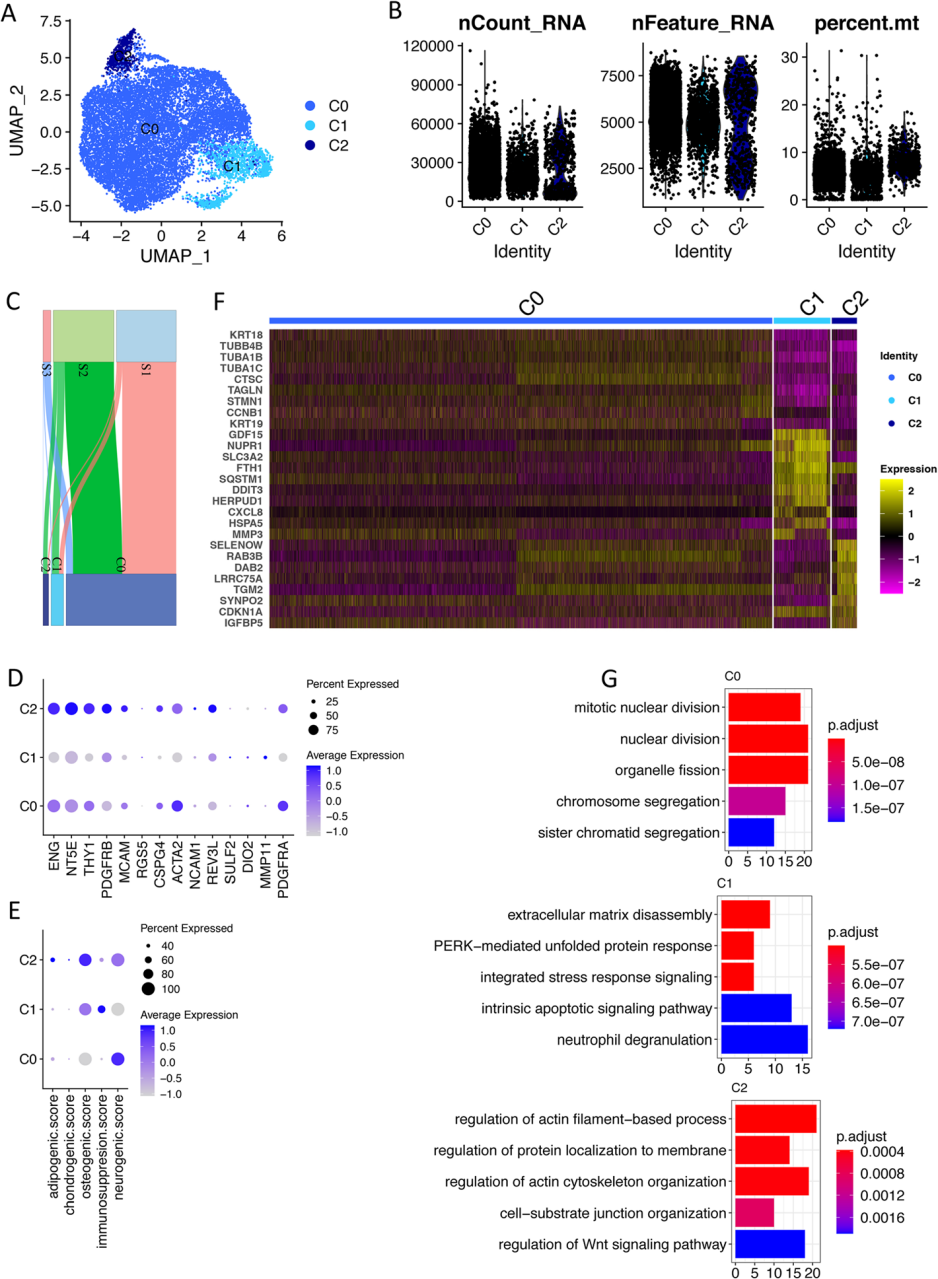
scRNA-seq reveals three sub-clusters in cultured secretory ePCs. **a** Uniform Manifold Approximation and Projection (UMAP) plot of the cellular sub-clusters. Single cells are color coded by cluster annotation. **b** Violin plot showing the distribution of number of UMIs, number of genes, and percentage of mitochondria reads of single cells in each sub-cluster. **c** Sankey diagram showing the contribution of cells from each sample to the sub-clusters. **d** Dot plot showing the expression of MSC markers, perivascular cell markers, smooth muscle cell markers, and stromal fibroblast cell markers across sub-clusters. Circle size indicates the percentage of cells in which the gene expression was detected. Fill color depicts the averaged normalized expression level of all cells within that sub-cluster. **e** Dot plot showing the score value of adipogenic, chondrogenic, osteogenic, and neurogeneic differentiation and immunomodulation potential across sub-clusters. The score for each cell for each term was firstly calculated by averaging the normalized-expression value of markers in each term. **f** Heat map of top upregulated genes in sub-clusters C0, C1, and C2. Sub-clusters were clearly separated. **g** Top significant enriched biological processes from gene ontology analysis based on upregulated genes identified in each sub-cluster. P.adjust, adjusted *p* value, value on *x* axis is the enrichment fold

Similar analysis approach was conducted for the 15,847 cultured secretory ePCs. Primary clustering identified 3 clusters with one of which showed low quality (Figure S4A, S4B). Clustering was re-conducted on the remaining cells (14,157) after exclusion of these low-quality cells. Finally, three stable clusters (C0, C1, C2) were identified with high quality (Fig. 4a, b) under the guidance of Silhouette score (Figure S4C). The contribution of each sample to each cluster is shown in Fig. 4c.

To understand the biological characteristics of the identified cell subpopulations, we firstly checked the expression of known MSC markers (ENG, NT5E, THY1), perivascular cell markers (PDGFRB, MCAM, RGS5, CSPG4), smooth muscle cell markers (ACTA2, ALPL, NCAM1), and stromal fibroblast cell markers (REV3L, SULF2, DIO2, MMP11, PDGFRA). The results showed that C0 and C2 expressed higher level of MSC markers, perivascular cell markers, smooth muscle cell markers, and stromal fibroblast marker PDGFRA when compared to C1 (Fig. 4d, Figure S4D). Same to the cultured menstrual ePCs, independent clustering on C0/C2 cluster could not further identify sub-clusters that clearly represented MSCs, perivascular cells, smooth muscle cells, and stromal fibroblast cells (data not shown). We further checked the lineage differentiation ability defined by lineage marker expression (Table S2). C0 and C2 exhibited higher neurogenic differentiation ability, C2 additionally exhibited higher osteogenic differentiation ability, and C1 exhibited higher immunomodulation ability which was similar to that of menstrual C1 (Fig. 4e, Figure S4E).

In addition to the investigation on known cell markers, we also identified upregulated genes (UGs) in all the clusters. The top UGs clearly separated C0, C1, and C2 (Fig. 4f. Gene ontology (GO) enrichment analysis on UGs in C0 identified biological processes related to cell division suggesting its proliferative identity (Fig. 4g). UGs in C1 enriched out biological processes including extracellular matrix disassembly, stress response, and apoptotic signaling (Fig. 4g). The GO enrichment results together with high expression of stress-related genes and senescence-associated inflammatory mediator in C1 suggested the senescent cell identity of this cell subpopulation (Fig. 4f, g). Indeed, the genes involved in DNA-damage-induced senescence preferentially expressed in C1 cells (Figure S4F). Unlike C0 and C1, UGs in C2 enriched out biological processes related to actin filament and protein localization.

In summary, for the cultured secretory ePCs, we revealed a main subpopulation C0 (12,179 cells) which manifested characteristics of MSC, perivascular cells, smooth muscle cells, and stromal fibroblast cells by expressing respective known cell identity markers. We also identified two small subpopulations C1 (1370) and C2 (608). C1 was annotated as a group of senescent cells based on its gene expression profile and GO enrichment results. Whether C1 was a technical induced subpopulation by mechanistic single-cell isolation or a natural subpopulation emerged from culturing required further investigation. Like C0, C2 also simultaneously expressed MSC markers, perivascular cell markers, smooth muscle cell markers, and stromal fibroblast markers. However, CDKN1A, a gene associated with DNA-damage-induced cell cycle arrest expressed higher in both C1 and C2 than that in C0 (Fig. 4f, Figure S4G). Thus, we speculated that C2 might be a subset of cells that resembled C0 but displayed elevated senescent properties.

### Cell similarity identified for cultured menstrual and secretory ePCs by MetaNeighbor analysis

**Fig. 5:**
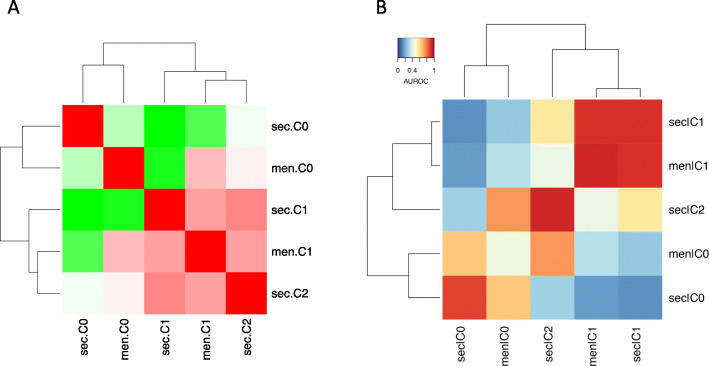
Cell identity replicability analysis identifies similarity between cultured menstrual and secretory ePCs. **a** Heat map of the Pearson correlation coefficient between sub-clusters from menstrual and secretory. All the values were scaled. sec., secretory; men., menstrual. Pearson correlation coefficient of each comparison: men.C0-sec.C0: 0.91; men.C0-sec.C1: 0.86; men.C0-sec.C2: 0.94; men.C1-sec.C0: 0.88; men.C1-sec.C1: 0.95; men.C1-sec.C2: 0.95. **b** Heatmap of the mean AUROC (area under the receiver operator characteristic curve) scores for identified sub-clusters between menstrual and secretory ePCs. AUROC scores of each comparison: men.C0-sec.C0: 0.65; men.C0-sec.C1: 0.27; men.C0-sec.C2: 0.74; men.C1-sec.C0: 0.18; men.C1-sec.C1: 0.89; men.C1-sec.C2: 0.43

Respective analysis for the cultured menstrual and secretory ePCs identified similar cell subpopulations by expressing limited number of known markers. To determine the relationship among subpopulations of cultured ePCs from different menstrual-cycle stages in a more sophisticated way, we utilized another two methods. One method was to calculate the Pearson correlation coefficient between average gene expressions of different subpopulations. The other method was to determine cell-cell similarity of subpopulations at single level using MetaNeighbor [27]. The Pearson correlation coefficient analysis revealed the higher similarity between menstrual C0 and secretory C0, menstrual C1 and secretory C1 (Fig. 5a). Secretory C2 exhibited comparable similarity to menstrual C0 (Pearson correlation coefficient 0.94) and C1 (Pearson correlation coefficient 0.95) (Fig. 5a). The similarity between menstrual C0 and secretory C0, menstrual C1 and secretory C1 were further supported by MetaNeighbor analysis (Fig. 5b). Secretory C2 demonstrated higher similarity to menstrual C0 (MetaNeighbor AUROC value 0.74) than C1 (MetaNeighbor AUROC value 0.43). In previous known marker expression analysis, secretory C2 was also similar to secretory C0 by expressing MSC markers, perivascular cell markers, smooth muscle cell markers, and stromal fibroblast cell markers. In summary, under the culture condition, similar cell composition of ePCs from menstrual phase and secretory phase was found.

### Cultured ePCs do not corresponds to primary ePCs by single-cell analysis

**Fig. 6:**
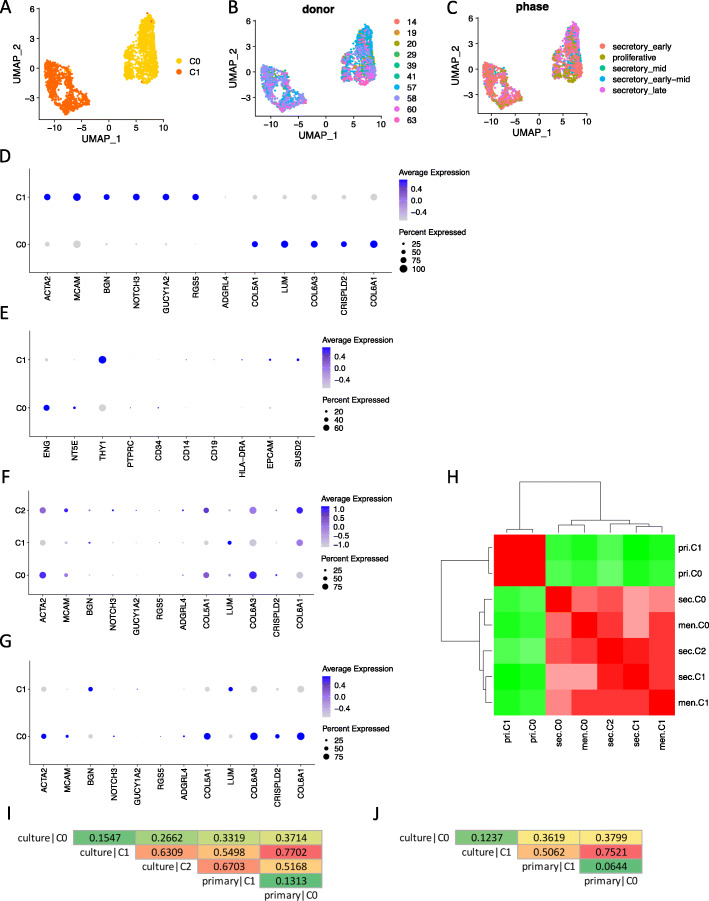
ePCs undergoes extensive changes upon culture. **a** UMAP plot of the cellular sub-clusters identified from primary PDGFRB^+^MCAM^+^ endometrial cells. Single cells are color coded by cluster annotation. **b** UMAP plot showing the distribution of cells color coded by donor. **c** UMAP plot showing the distribution of cells color coded by menstrual phases assigned by the published study. **d** Dot plot showing the expression of perivascular cell markers, and stromal fibroblast cell markers used in the published study across sub-clusters of primary PDGFRB^+^MCAM^+^ endometrial cells. **e** Dot plot showing the expression of MSC definition markers, epithelia cell marker EPCAM, and another eMSC enrichment marker SUSD2 across sub-clusters of primary PDGFRB^+^MCAM^+^ endometrial cells. **f** Dot plot showing the expression of perivascular cell markers, and stromal fibroblast cell markers used in the published study across sub-clusters of secretory ePCs. **g** Dot plot showing the expression of perivascular cell markers, and stromal fibroblast cell markers used in the published study across sub-clusters of menstrual ePCs. **h** Heat map of the Pearson correlation coefficient between sub-clusters from menstrual, secretory ePCs, and primary PDGFRB^+^MCAM^+^ endometrial cells. **i** AUROC scores between sub-clusters of secretory ePCs and of primary PDGFRB^+^MCAM^+^ endometrial cells. The scores are color-scaled. **j** AUROC scores between sub-clusters of menstrual ePCs and of primary PDGFRB^+^MCAM^+^ endometrial cells. The scores are color-scaled

ePCs cultured in vitro manifested properties of MSCs with clonogenic and multiple differentiation abilities. It was also reported that cultured ePCs could produce endometrial stromal-like tissues in vivo after re-introducing into mouse [30]. However, the relationship between MSCs in vitro and perivascular cells in vivo is still a debating area [31, 32]. To explore the situation between cultured ePCs and uncultured primary ePCs, we for the first time performed comparison analysis at single-cell level. We utilized a public dataset (GSE111976) in which primary endometrial cells from healthy ovum donors were profiled using 10X Genomics technology [33]. Only cells simultaneously expressing PDGFRB and MCAM (UMI count > 1) were selected out from the public dataset for further analysis. The same single-cell analysis approach in this study was applied to the primary PDGFRB^+^MCAM^+^ endometrial stromal cells. The results showed that two separated clusters were identified (Fig. 6a) within which cells showed no biased distribution either across donors (Fig. 6b) or menstrual phases (Fig. 6c) assigned by the published study. Perivascular cell markers and stromal fibroblast cell markers used in the published study were differentially expressed in the two clusters showing the cell identity of C0 as stromal fibroblast cells, and C1 as perivascular cells (Fig. 6d, Figure S5A). Interestingly, MCAM, whose protein was detected only in perivascular and endothelial cells, was also expressed in the stromal fibroblast cell cluster C0 (Figure S5A). Whether this level of RNA expression could represent protein expression required further investigation. To compare cultured ePCs and primary ePCs, we firstly checked the MSC marker expression in primary ePCs. The results showed that primary ePCs did not consistently expressed the three positive MSC markers ENG, NT5E, and THY1 indicating that the MSC characteristics of ePCs might emerge during culture (Fig. 6e, Figure S5B). We further compared subpopulations of cultured ePCs to primary subpopulations C0 (stromal fibroblasts) and C1 (perivascular cells). The expression of perivascular cell markers (ACTA2, MCAM) and stromal fibroblast cell markers (COL5A1, COL6A3) were simultaneously expressed in C0 and C2 of cultured secretory ePCs (Fig. 6f, Figure S5C), as well as in C0 of cultured menstrual ePCs (Fig. 6g, Figure S5D). Pearson correlation analysis showed that primary subpopulations were distinct from cultured subpopulations (Fig. 6h) which was reasonable because these were two datasets generated from different laboratories. We further utilized MetaNeighbor which could evaluate cell-cell similarity across single-cell sequencing datasets to determine the relationship among different subpopulations. The results revealed that both secretory C1 and menstrual C1 were similar to primary C0 (stromal fibroblast) with AUROC value greater than 0.7 indicating their non-ePC identity (Fig. 6i, j). From previous analysis, we could know secretory C1 and menstrual C1 were similar subpopulations’ characteristic of senescence. In addition, they were losing expression of perivascular cell markers and gaining expression of stromal fibroblast markers further supporting the MetaNeighbor result. The main subpopulations of secretory (C0) and menstrual (C0) ePCs demonstrated little similarity to either primary stromal fibroblasts or perivascular cells indicating their culture uniqueness (Fig. 6i, j). To demonstrate that the results are not dataset-sensitive, we further utilized an independent public dataset (E-MTAB-6701) in which decidual tissue from elective terminations of normal pregnancy were subjected to scRNA-seq [34]. Cells expressing both PDGFRB and MCAM (UMI count > 0) were selected out and similar analyses were performed on them. Two clusters (C0, C1) were identified (Figure S6A) which respectively resembled stromal fibroblasts and perivascular cells identified from public dataset GSE111976 with AUROC scores over 0.9 (Figure S6B). Marker gene expression confirmed their cell identity as stromal fibroblast and perivascular cells (Figure S6C). Similar to previous finding, MSC markers were not consistently expressed in perivascular cell cluster C1 (Figure S6D). Cell identity analysis also showed that culture cell identity was different from either the primary fibroblast or perivascular cells (Figure S6E, S6F).

In this part, by comparing cultured ePCs from secretory and menstrual phases to primary PDGFRB^+^MCAM^+^ (CD140b^+^CD146^+^) endometrial stromal cells, we revealed that cultured ePCs no longer corresponded to primary ePCs underscoring the importance of in vivo microenvironment. Moreover, primary ePCs do not express all positive MSC markers suggesting the possibility that the MSC characteristics of cultured ePCs might emerge from the culturing process. Whether in situ ePCs possess the multipotency of MSC in vitro requires further study.

## Discussion

Endometrial MSCs have been proposed to play critical roles in the cyclic regeneration of human endometrium [10]. Isolation of coexpressing CD140b and CD146 endometrial perivascular cells (ePCs) show characteristics of MSC in terms of clonogenic, multiple differentiation, and immunomodulation ability in vitro [13]. Like MSCs, CD140b and CD146 double-positive ePCs cultured in vitro have been deemed as a promising source for cell therapy because of its easier accessibility. However, before application to cell transplantation, the cellular heterogeneity which may affect clinical outcome, requires extensive investigation. In addition to cell transplantation using cultured ePCs, in vivo ePCs have also been regarded as alternative cell source for stem cell regeneration with in situ activation [10, 11]. However, the relationship between cultured ePCs and primary ePCs remains unknown.

In this study, we conducted a comprehensive single-cell study on CD140b^+^CD146^+^ ePCs from different menstrual phases which aims to study their cellular heterogeneity, and relationship to primary CD140b^+^CD146^+^ ePCs. In single-cell analysis, one caution is to remove batch effect before exploration on the biological characteristics. In this study, by evaluation on the three benchmark methods on batch-effect removal, we found out that Seurat v3 performed the best when different versions of chemistry reagents were used, while Harmony achieved the best performance when the same versions of chemistry reagents were used under the scenario of biological similar samples. This information would be useful for those who are utilizing public datasets generated from different versions of chemistry reagents of 10X Genomics.

Utilizing the high-quality single-cell data, we separately analyzed cultured ePCs from menstrual and secretory phases. Two clusters for cultured menstrual ePCs and three clusters for cultured secretory ePCs were identified. Correlation and cell identity classification analysis showed that no menstrual-cycle-stage-specific cluster was identified suggesting the similar cell composition for cultured secretory and menstrual ePCs. Previous report also demonstrated that no molecular changes were observed for ePCs from different menstrual phases [12]. Gene expression analysis revealed that the small cell subpopulation undergone DNA-damage-induced senescence which might be a common cellular fate for eMSCs or a group of stressed cells caused by the single-cell mechanistic isolation. In addition, the main cell subpopulation exhibited potentials of MSCs, perivascular cells, smooth muscle cells, and stromal fibroblasts by expressing markers in each cell category. The expression of markers for different cell types indicated the changed phenotype of ePCs upon culture. This speculation was further supported by the comparison analysis to primary PDGFRB^+^MCAM^+^ ePCs at single-cell level. We found that RGS5, which was highly expressed in primary ePCs, no longer expressed in cultured ePCs (Fig. 6f, g). RGS5 was reported to be a functional gene in regulating vessel wall remodeling [35]. Whether RGS5 plays key roles in primary ePCs is not clear. But the total loss of RGS5 upon culture implies the inability of cultured ePCs to represent primary ePCs in physiological condition. On the contrary, MSC markers that were expressed in culture ePCs were not consistently expressed in primary ePCs. Additional cell-identity replicability analysis showed the main subpopulation in culture was unique which was unlike primary ePCs or primary endometrial stromal fibroblasts. All these results indicated the change of ePCs upon culture and underscored the importance of in vivo microenvironment in maintaining cell identity. It also challenges the view that in vivo ePCs contribute to the regeneration of endometrium. Indeed, in other organs, the contribution of PCs to tissue regeneration still remains controversial. Crisan et al. showed that MSCs were pericytes [36]. Dellavalle et al. demonstrated the contribution of pericytes to muscle regeneration [37, 38]. However, Camboa et al. reported that pericytes could not produce somatic cells in multiple organs under aging and injury [39]. Therefore, the contribution of primary ePCs to endometrial regeneration in vivo requires further investigation. On one hand, although observation of distinct molecular profiles of cultured ePCs from primary ePCs discourages the use of pericytes within endometrium as endogenous progenitors for regeneration, it does not challenge the beneficial effects of transplantation of cultured ePCs for cell therapy. On the other hand, the cellular identity discrepancy between cultured ePCs and primary ePCs reveals the importance of in vivo microenvironment.

## Conclusion

In summary, we report the first single-cell sequencing study on eMSCs based on a large offset of cells. In addition, cells were obtained from several donors at different menstrual phases, ensuring the comprehensiveness. Undoubtedly, this large cell atlas of human cultured CD140b^+^CD146^+^ ePCs provides an essential resource for a better understanding into the nature of eMSCs. It also could be utilized to develop guidance for the production of homogeneous eMSCs for cell therapy and to find out microenvironmental factors maintaining their cell identity. In the future, more work should be done to experimentally validate the findings based on single-cell analysis and to determine the generalization of the present observations in other eMSC population.

## Supplementary Information

**Additional file 1:** Table S1. Table S2. Figure S1. Figure S2. Figure S3. Figure S4. Figure S5. Figure S6.

## Data Availability

The data that support the findings of this study have been deposited in the Gene Expression Omnibus (GEO) database with accession number GSE149651. The publicly available datasets of primary endometrial cells was downloaded from GEO database with accession number GSE111976 and from the European Molecular Biology Laboratory’s European Bioinformatics Institute (EMBL-EBI) database with accession number E-MTAB-6701. All data analyzed during this study have been included.

## Abbreviations

AUROC: Area under the receiver operator characteristic curve
eMSCs: Endometrial mesenchymal stromal/stem cells
ePC: Endometrial perivascular cells
GO: Gene ontology
KNN: K-nearest neighbor
MSCs: Mesenchymal stromal/stem cells
PCA: Principle component analysis
scRNA-seq: Single-cell RNA sequencing
SNN: Shared nearest neighbor
UGs: Upregulated genes
UMAP: Uniform Manifold Approximation and Projection
UMI: Unique molecular identifiers
