# Correction: Single-cell RNA sequencing of cultured human endometrial CD140b^+^CD146^+^ perivascular cells highlights the importance of in vivo microenvironment

**DOI:** 10.1186/s13287-022-02872-6

**Published:** 2022-05-05

**Authors:** Dandan Cao, Rachel W. S. Chan, Ernest H. Y. Ng, Kristina Gemzell-Danielsson, William S. B. Yeung

**Affiliations:** 1 Shenzhen Key Laboratory of Fertility Regulation, Reproductive Medicine Center, The University of Hong Kong - Shenzhen Hospital, Shenzhen, China; 2 Department of Obstetrics and Gynaecology, LKS Faculty of Medicine, The University of Hong Kong, Pokfulam, Hong Kong, SAR China; 3 Department of Women’s and Children’s Health, Division of Obstetrics and Gynecology, Karolinska Institutet and Karolinska University Hospital, Solna, Sweden; 4 Department of Obstetrics and Gynecology, The University of Hong Kong, Pokfulam, Hong Kong, SAR China

## Correction: Stem Cell Research & Therapy (2021) 12:306 https://doi.org/10.1186/s13287-021-02354-1

The original article [1] noted two erroneous grant numbers which have both since been corrected.
