# Supplementary material for: Single-cell RNA sequencing of cultured human endometrial CD140b+CD146+ perivascular cells highlights the importance of in vivo microenvironment
Source: Stem Cell Res Ther. 2021 May 29;12:306. doi: 10.1186/s13287-021-02354-1 (PMC8164319; doi:10.1186/s13287-021-02354-1)
Supplement: Supplementary file 1 — Additional file 1: Table S1. Table S2. Figure S1. Figure S2. Figure S3. Figure S4. Figure S5. Figure S6. [file 13287_2021_2354_MOESM1_ESM.docx]

Supplementary Information

Single-cell RNA sequencing of cultured human endometrial CD140b^+^CD146^+^ perivascular cells highlights the importance of in vivo microenvironment

Dandan Cao, Rachel W.S. Chan, Ernest H.Y. Ng, Kristina Gemzell-Danielsson, William S.B. Yeung

**Table S1.** Sample information

| **ID** | **Age range** | **Phase** | **Description** | **Chemistry reagent version in 10X Genomics platform** |
| --- | --- | --- | --- | --- |
| M1 | 40-49 | Menstrual | Endometrial aspirates | v2 |
| M2 | 30-39 |  |  | v2 |
| M3 | 30-39 |  |  | v3 |
| S1 | 40-49 | Secretory | Full-thickness endometrium from hysterectomy | v3 |
| S2 | 50-59 |  |  | v3 |
| S3 | 40-49 |  |  | v2 |

**Table S2**. Genes used to calculate scores for different categories

| **Categories** | **Genes** |
| --- | --- |
| Adipogenic | *PPARG, RHOA, CEBPA, CEBPB, LEPR* |
| Chondrogenic | *HAT1, ITGAX, KAT2B, SOX9, ACAN, COL2A1, MMP13, CSPG4, BMP4, TGFB1, TGFB3, BMP6, KDR, MSX1, MSX2, BMP2, PRRX1* |
| Osteognic | *ALPL, IBSP, SP7, BGLAP, BMP7, COL1A1, FGF10, HDAC1, PTK2, SMURF1, SMURF2, TBX5, RUNX2, FGF9, BMP4, TGFB1, TGFB3, BMP6, KDR, MSX1, MSX2, BMP2, PRRX1* |
| Neurogenic | *NES, GFAP, PAX6, SOX2, NCAM1, NEUROD1, VIM* |
| Immunosuppresion | *CCL2, CSF3, VEGFA, IL7* |

**
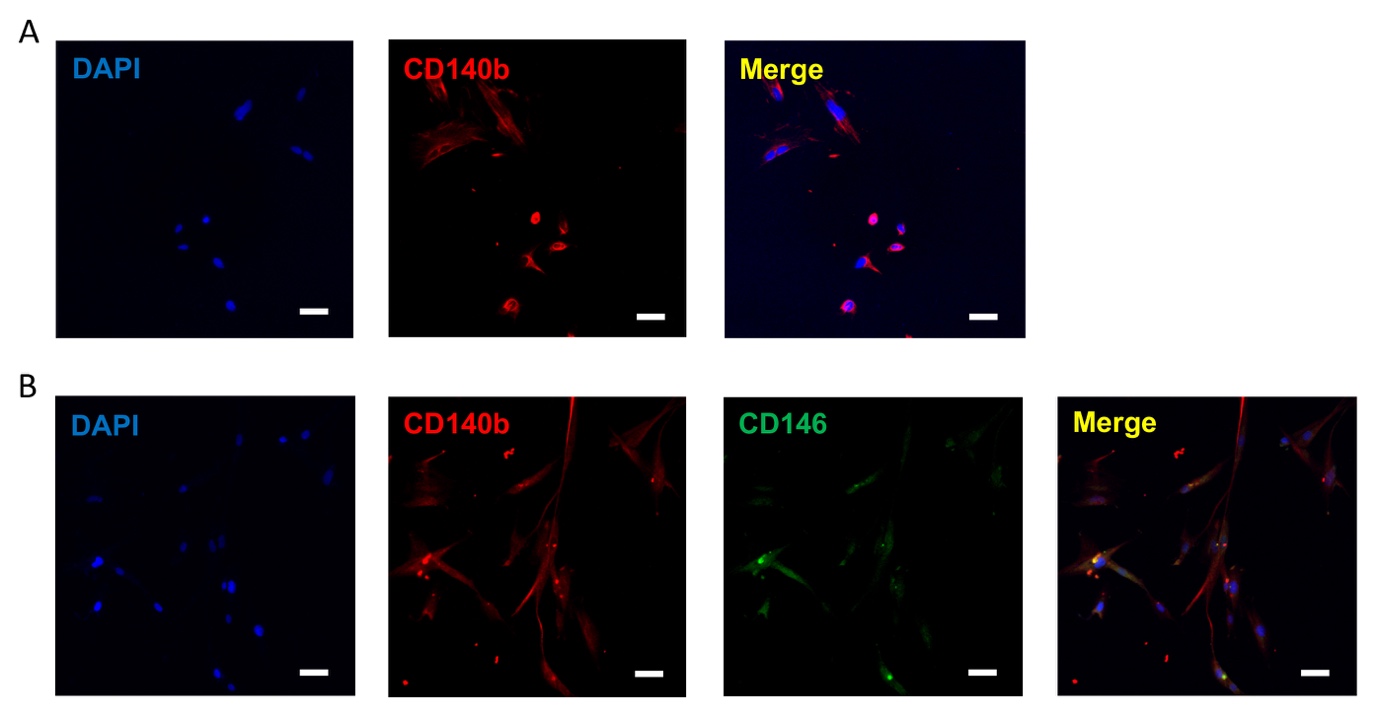
**

**Figure S1. Immunofluorescent staining of CD140b and CD146 on endometrial stromal cells after microbeads isolation.** (**A**) The surface marker CD140b (red) on endometrial stromal cells was confirmed by immunofluorescence after CD140b^+^ microbeads selection. (**B**) All cells co-expressed CD140b (red) and CD146 (green) after the second CD146^+^ microbeads isolation. DAPI nuclear stain (blue). Scale bar: 100 µM.

**
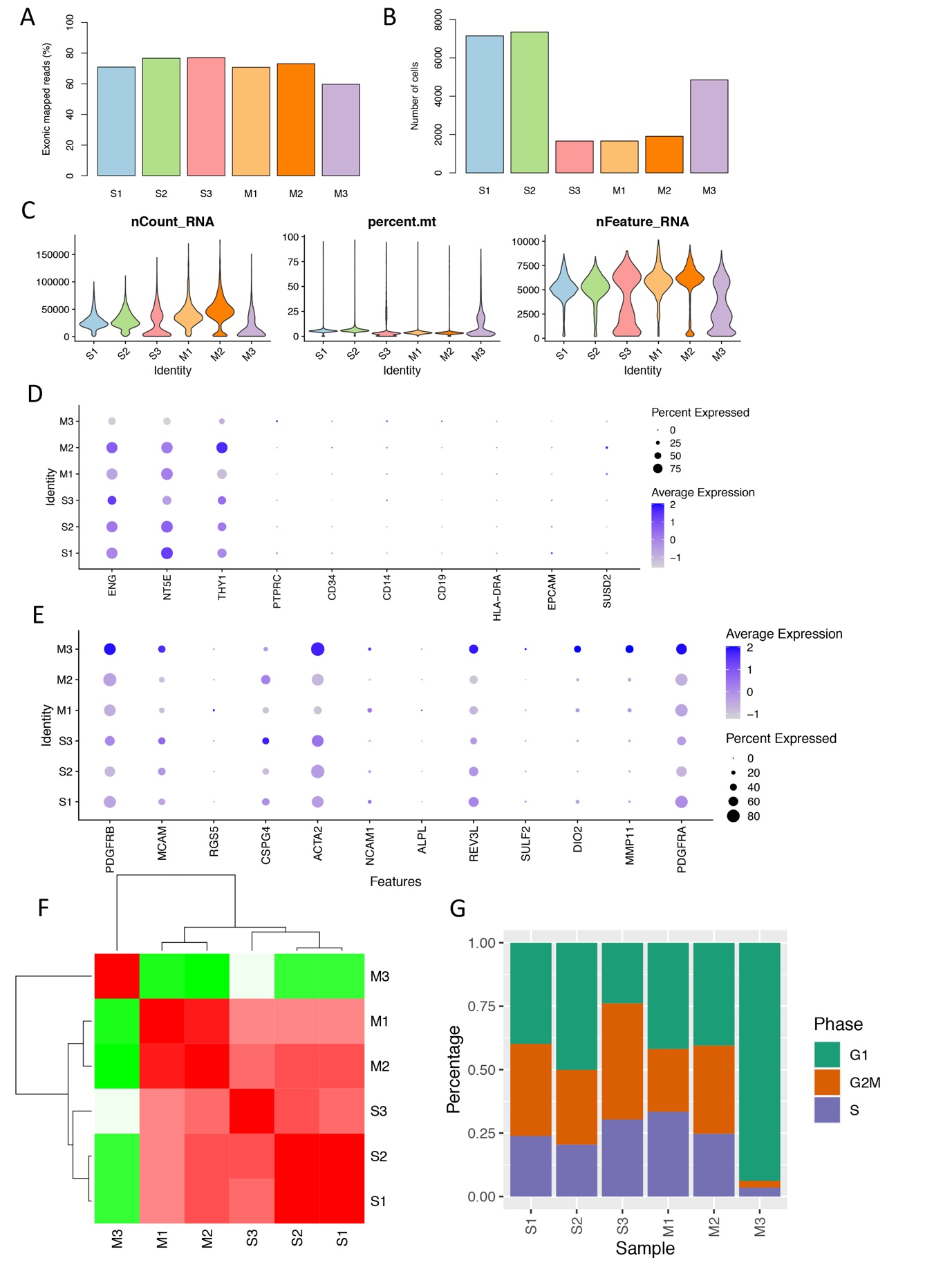
**

**Figure S2. Quality control of scRNA-seq data**. (**A**) Exonic mapping rate of each sample. (**B**) Detected cell number by CellRanger v3 of each sample. (**C**) Violin plot showing the distribution of number of UMIs, number of genes, percentage of mitochondria reads of single cells in each sample. (**D**) Dot plot showing the expression of MSC markers (both positive and negative), epithelial cell marker EPCAM and another eMSC enrichment marker SUSD2 across each sample. Circle size indicates the percentage of cells in which the gene expression was detected. Fill color depicts the averaged normalized expression level of all cells within that sample. (**E**) Dot plot showing the expression of perivascular cell marker, smooth muscle cell marker, and stromal fibroblast cell markers across each sample. (**F**) Heat map of the Pearson correlation coefficient between samples. All the values were scaled. (**G**) Bar plot showing the percentage of cell distribution on cell cycle phases for each sample.

**
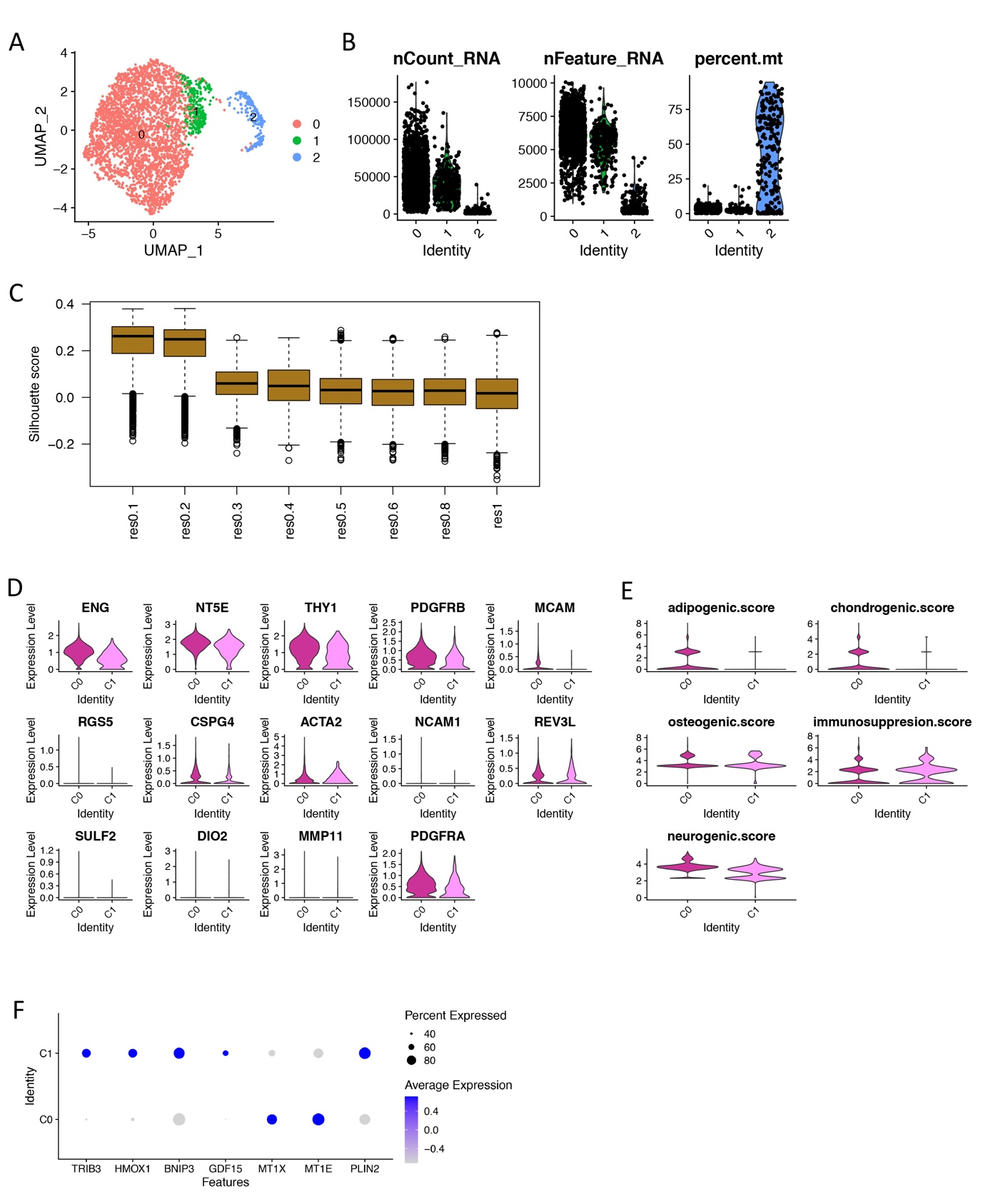
Figure S3. Identification of sub-clusters in cultured menstrual ePCs**. (**A**) Uniform Manifold Approximation and Projection (UMAP) plot of the primary cellular sub-clusters. Single cells are color coded by cluster annotation. (**B**) Violin plot showing the distribution of number of UMIs, number of genes, percentage of mitochondria reads of single cells in each sample. (**C**) Box plot of Silhouette scores for sub-clusters identified from different resolutions. (**D**) Violin plot showing the expression of MSC markers, perivascular cell markers, smooth muscle cell markers and stromal fibroblast cell markers across final sub-clusters. (**E**) Violin plot showing the score value of adipogenic, chondrogenic, osteogenic, neurogeneic differentiation and immunomodulation potential across sub-clusters. The score for each cell for each term was firstly calculated by averaging the normalized-expression value of markers in each term. (**F**) Dot plot showing the expression of markers associated with DNA-damage induced senescence across each sub-cluster.

**
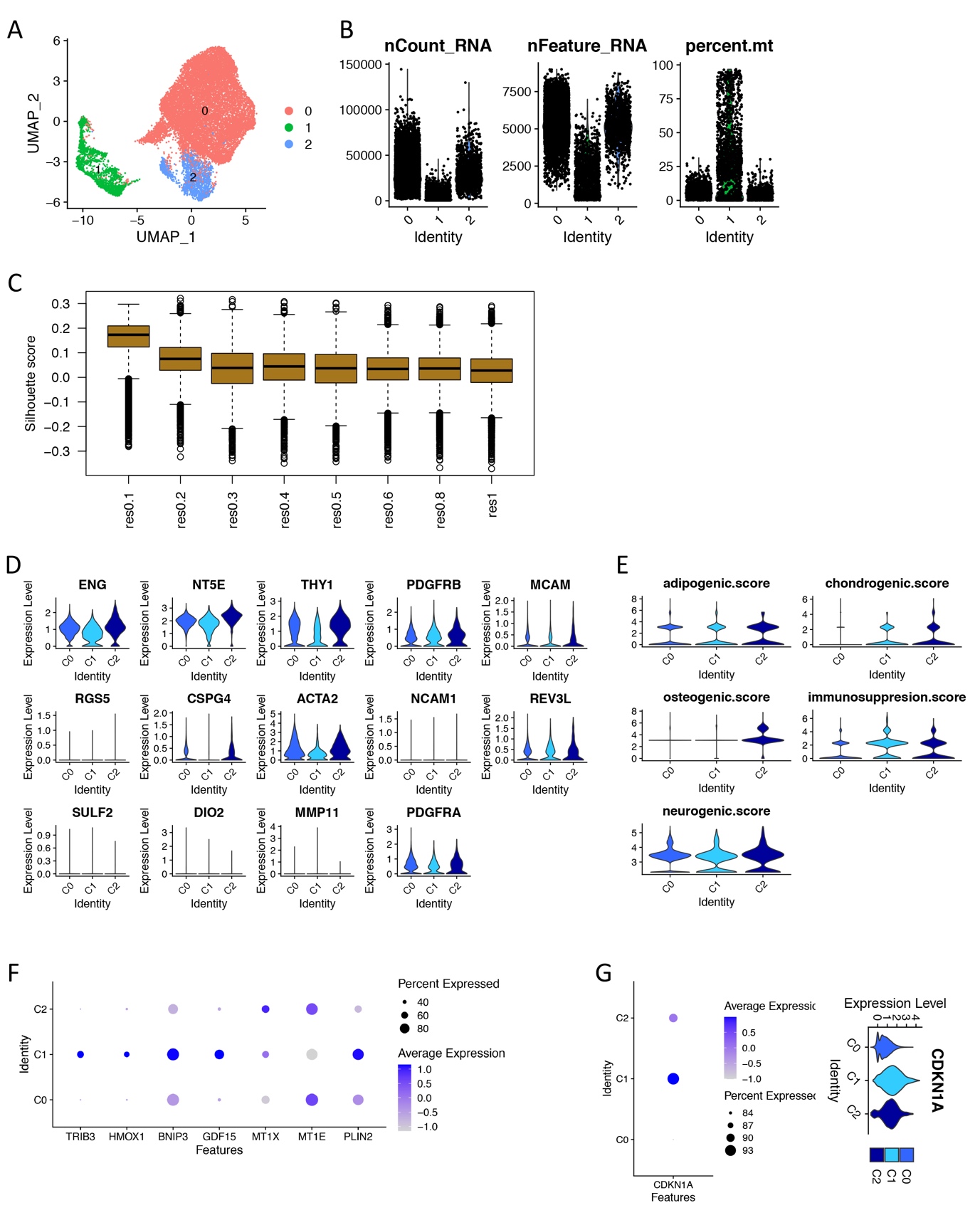
**

**Figure S4. Identification of sub-clusters in cultured secretory ePCs**. (**A**) Uniform Manifold Approximation and Projection (UMAP) plot of the primary cellular sub-clusters. Single cells are color coded by cluster annotation. (**B**) Violin plot showing the distribution of number of UMIs, number of genes, percentage of mitochondria reads of single cells in each sample. (**C**) Box plot of Silhouette scores for sub-clusters identified from different resolutions.(**D**) Violin plot showing the expression of MSC markers, perivascular cell markers, smooth muscle cell markers and stromal fibroblast cell markers across final sub-clusters. (**E**) Violin plot showing the score value of adipogenic, chondrogenic, osteogenic, neurogeneic differentiation and immunomodulation potential across sub-clusters. The score for each cell for each term was firstly calculated by averaging the normalized-expression value of markers in each term. (**F**) Dot plot showing the expression of markers associated with DNA-damage induced senescence across each sub-cluster. (**G**) Dot plot and violin plot showing the expression of CDKN1A across each sub-cluster.

**
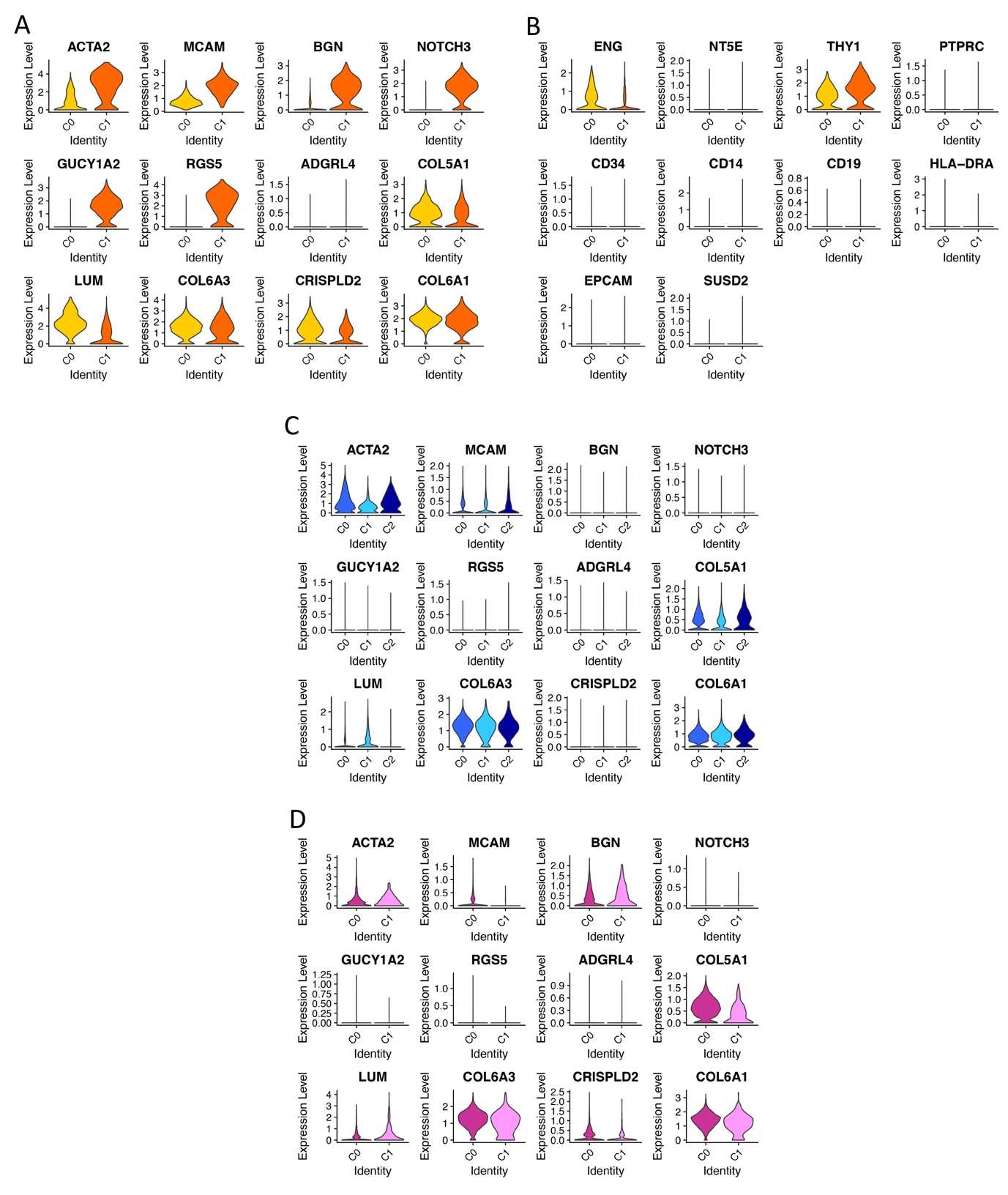
**

**Figure S5. Gene expression in sub-clusters of cultured menstrual and secretory ePCs, as well as primary endometrial cells (GSE111976).** (**A**) Violin plot showing the expression of perivascular cell markers, and stromal fibroblast cell markers used in the published study across sub-clusters of primary PDGFRB^+^MCAM^+^ endometrial cells. (**B**) Violin plot showing the expression of MSC definition markers, epithelia cell marker EPCAM, and another eMSC enrichment marker SUSD2 across sub-clusters of primary PDGFRB^+^MCAM^+^ endometrial cells. (**C**) Violin plot showing the expression of perivascular cell markers, and stromal fibroblast cell markers used in the published study across sub-clusters of cultured secretory ePCs. (**D**) Violin plot showing the expression of perivascular cell markers, and stromal fibroblast cell markers used in the published study across sub-clusters of cultured menstrual ePCs.

**
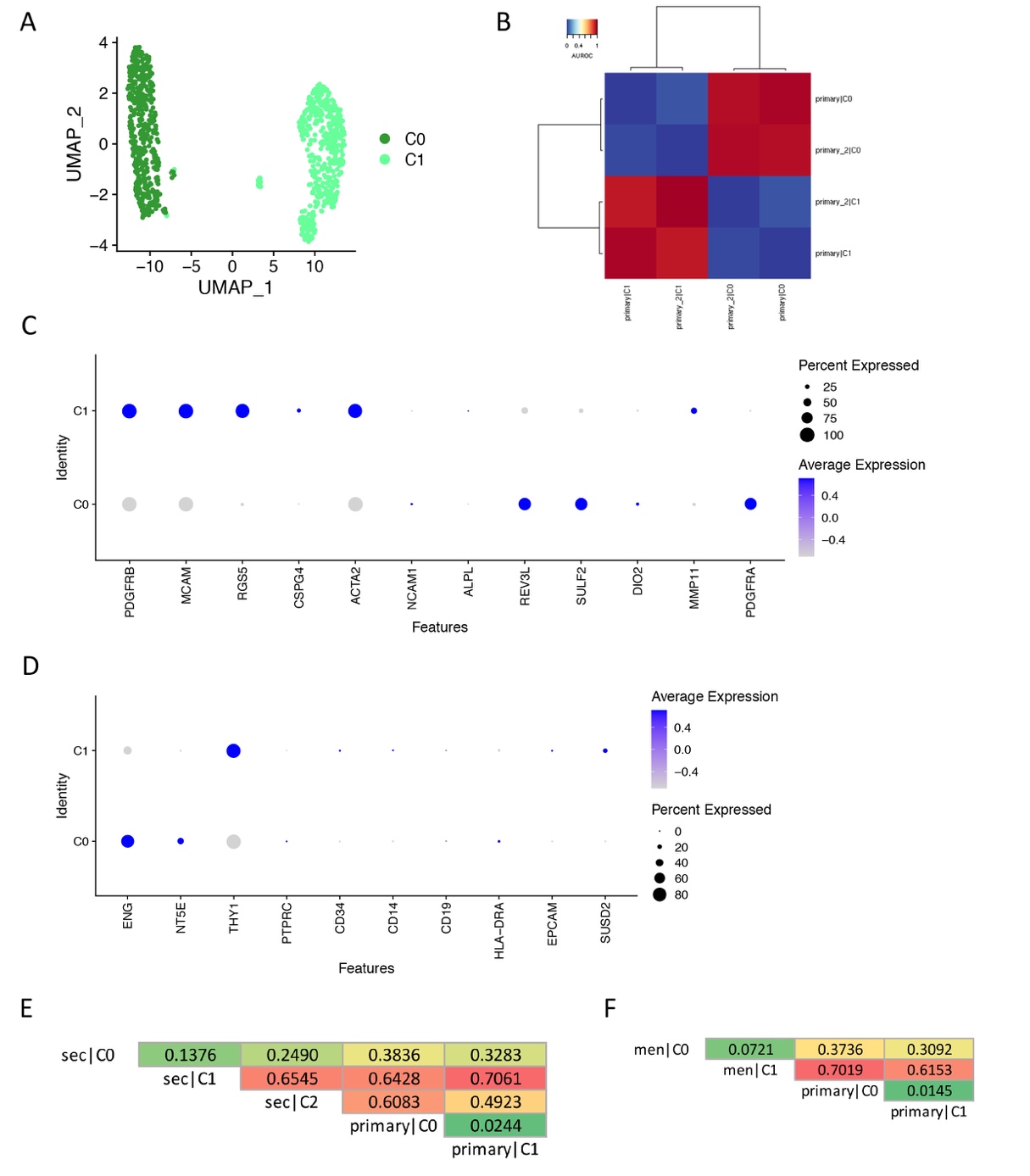
**

**Figure S6. Comparison analysis of culture ePCs and primary endometrial cells from public dataset (E-MTAB-6701).** (**A**) UMAP plot of the cellular sub-clusters identified from primary PDGFRB^+^MCAM^+^ endometrial cells. Single cells are color coded by cluster annotation. (**B**) Heatmap of the mean AUROC scores for identified sub-clusters between different public datasets (primary for GSE111976**,** primary_2 for E-MTAB-6701). AUROC scores of comparison: C0-C0: 0.96; C1-C1: 0.95. (**C**) Dot plot showing the expression of perivascular cell markers, and stromal fibroblast cell markers across sub-clusters of primary PDGFRB^+^MCAM^+^ endometrial cells. (**D**) Dot plot showing the expression of MSC definition markers, epithelia cell marker EPCAM, and another eMSC enrichment marker SUSD2 across sub-clusters of primary PDGFRB^+^MCAM^+^ endometrial cells. (**E**) AUROC scores between sub-clusters of secretory ePCs and of primary PDGFRB^+^MCAM^+^ endometrial cells. The scores are color-scaled. (**F**) AUROC scores between sub-clusters of menstrual ePCs and of primary PDGFRB^+^MCAM^+^ endometrial cells. The scores are color-scaled.
